# How Have the COVID-19 Pandemic and Market Sentiment Affected the FX Market? Evidence from Statistical Models and Deep Learning Algorithms

**DOI:** 10.1007/s44196-023-00194-w

**Published:** 2023-02-25

**Authors:** Hang (Robin) Luo, Xiaoyu Luo, Shuhao Gu

**Affiliations:** 1 DVC Office, Wuhan College, Wuhan, 430212 People’s Republic of China; 2 School of Economics, Xihua University, Chengdu, 610039 People’s Republic of China

**Keywords:** COVID-19, Deep learning, Ensemble model, Exchange rate, Sentiment

## Abstract

This paper attempts to investigate the impact of the COVID-19 pandemic and market sentiment on the dynamics of USD/JPY, GBP/USD, and USD/CNY. We compose the market sentiment variable and incorporate the newly confirmed COVID-19 cases and sentiment variable into the traditional exchange rate forecasting model. We find that confirmed COVID-19 cases and sentiment variables in the US, Japan, UK, and China in the period of January 23rd, 2020 to September 14th, 2021 are significant in explaining the bilateral exchange rate movement. Recurrent neural network (RNN) and long short-term memory (LSTM) models outperform the other deep learning models and vector autoregressive (VAR) model in forecasting the bilateral exchange rate movement during the COVID-19 pandemic period. Further analysis using high-frequency intraday data and ensemble models shows that ensemble models significantly improve the accuracy of exchange rate prediction, as they are better at coping with the nonlinear and nonstationary features of exchange rate time series.

## Introduction

The forex market is very sensitive to new economic or financial market information, as this information has a great influence on the international trade balance, interest rates, and eventually supply and demand. In particular, the exchange rate demonstrates extreme variations during the war, natural disaster, and crisis periods [6, 15]. The COVID-19 pandemic led to the collapse of many markets and a global economic plummet [1, 53]. Given the long-lasting economic consequences of the unprecedented COVID-19 pandemic, it is critical to understand its effects on market sentiment and exchange rate movement [32, 36, 40].

It is commonly believed that the impact of the pandemic on financial markets is prominent. Many major stock markets around the world lost almost 20% of their initial value during the COVID-19 pandemic [21, 31]. Major monetary authorities such as the European Central Bank (ECB) and the Federal Reserve Bank (FED) announced generous stimulus packages to stimulate their economy. ECB and FED set their interest rate targets close to zero at the same time to minimize the effects of lockdowns and panic on overall economic activity [17, 53]. As a result, it is apparent that the fundamental determinants of exchange rate dynamics have been substantially impacted by the COVID-19 pandemic [3, 39, 41].

In this context, the present study attempts to shed light on the research question that arises, which can be formulated as follows: What is the impact of the COVID-19 pandemic and market sentiment on the dynamics of bilateral exchange rates? To answer this question, we compose the market sentiment variable and incorporate the newly confirmed COVID-19 cases and sentiment variable into the traditional exchange rate forecasting model.

We find that newly confirmed COVID-19 cases and sentiment variables in the US, Japan, UK, and China in the period of January 23rd to September 14th, 2021 are significant in explaining the bilateral exchange rate movement. Deep learning models and the VAR model are then used to forecast the exchange rate return. The results of the predicted exchange rate returns show that the incorporation of the market sentiment indicator enhances predictive power. The RNN and LSTM models outperform the other deep learning models and VAR model in forecasting the bilateral exchange rate movement.

Further analysis using high-frequency intraday data and ensemble models shows that ensemble models significantly improve the accuracy of exchange rate prediction, as they are better at coping with the nonlinear and nonstationary features of exchange rate time series. The ensemble models integrating the statistical models and deep learning algorithms outperform the individual econometric model or individual deep learning model in exchange rate forecasting.

This paper is structured as follows: Sect. 2 presents a review of the literature. Section 3 describes the methodology used. Section 4 presents the empirical results during the COVID-19 pandemic period. Section 5 provides further analysis using high-frequency intraday data. Section 6 concludes the paper.

## Literature Review

Due to the enormous impact of the foreign exchange market on the micro- and macroeconomy, a variety of strategies have been developed to increase the accuracy of exchange rate forecasting [27, 42]. Statistical techniques and machine learning methods are the two types of strategies used by academics [45]. The statistical approach comprises vector autoregressive (VAR) [30], autoregressive integrated moving average (ARIMA) [33], generalized autoregressive conditional heteroskedasticity (GARCH) [28], and others that are simple to use and have been widely used in exchange rate forecasting.

Traditional statistical approaches cannot match the fluctuation well since the exchange rate time series is nonlinear and unstable, and forecast accuracy must be increased [54]. For instance, Liu et al. [30] analyzed the forecasting accuracy of different VAR models of the USD/JPY, USD/CAD, and USD/DEM exchange rates. Their results show that a monetary/asset model in a VAR representation does have forecasting value for some exchange rates. Lahmiri [29] employed traditional GARCH as a benchmark to anticipate USD/CAD and USD/EUR exchange rate volatility, however, the GARCH model’s prediction performance was not sufficient. The performance of ARIMA was studied by Zainuddin et al. [50], and the empirical results revealed that ARIMA model approximations were inadequate for complicated real-world issues.

However, the statistical technique produces disappointing results due to the limited distribution assumption’s limitations and the inability to capture nonlinear and nonstationary patterns. Machine learning is another technology that may be used to model nonlinear and nonstationary exchange rate data [4, 16]. Artificial neural networks (ANNs) have been shown to be superior to linear statistical approaches in exchange rate forecasting as a data-driven machine learning method due to their data-fitting capability [49].

Furthermore, support vector machine (SVM) and support vector regression (SVR) have been shown to have a low generalization error and the ability to learn from exchange rate time series [23, 37]. ANN and SVR have recently shown higher performance than classic statistical approaches, which have benefits in modeling intricate exchange rate data, thanks to the growth of machine learning [43]. The artificial neural network (ANN) is a sophisticated nonlinear prediction model that may attain arbitrary accuracy [20]. For forecasting exchange rates, Kiani and Kastens [26] utilized linear models and feedforward artificial neural networks, and the findings confirmed the usefulness of ANN.

For intraday market prediction, Evans et al. [14] suggested an ANN-based prediction model. The results showed that the proposed model had a high level of accuracy and might help in decision-making. Although studies have shown that ANN outperforms other machine-learning methods, it is an unstable machine-learning approach that tends to slip into local rather than global minima [12]. Since then, SVR has become one of the most commonly used approaches, as its structural risk reduction concept increases the model’s generalization ability.

Ince and Trafalis [23], for example, used SVR to estimate exchange rates across four datasets. The results indicated that SVR outperformed ANN in terms of exchange rate prediction. Furthermore, SVR is used for forecasting exchange rates based on reconstructed phase space, and empirical findings indicated that SVR had a greater prediction accuracy than back propagation neural network (BPNN) [22].

## Models

Referring to existing approaches for feature extraction followed by prediction, two deep learning techniques, namely, RNN and LSTM, and two integrated tree-based algorithms, namely, random forest (RF) and adaptive boosting (Adaboost), are used as base learners to forecast the bilateral exchange rate of USD/JPY, GBP/USD, and USD/CNY using daily data.

### RNN and LSTM

**Fig. 1:**
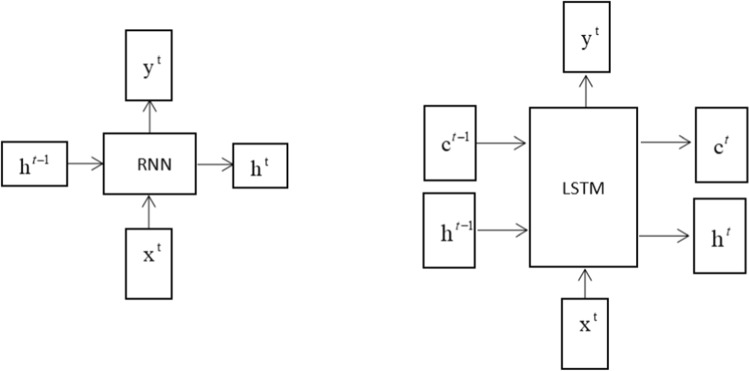
RNN and LSTM structure

RNN and LSTM are models commonly used in time series for nonlinear prediction problems [19], and LSTM more easily forms long-term dependence than the former [9–11. First, the two models are compared from the macro model operation steps, and the RNN and LSTM structure diagrams are as follows (Fig. 1).

**Fig. 2:**
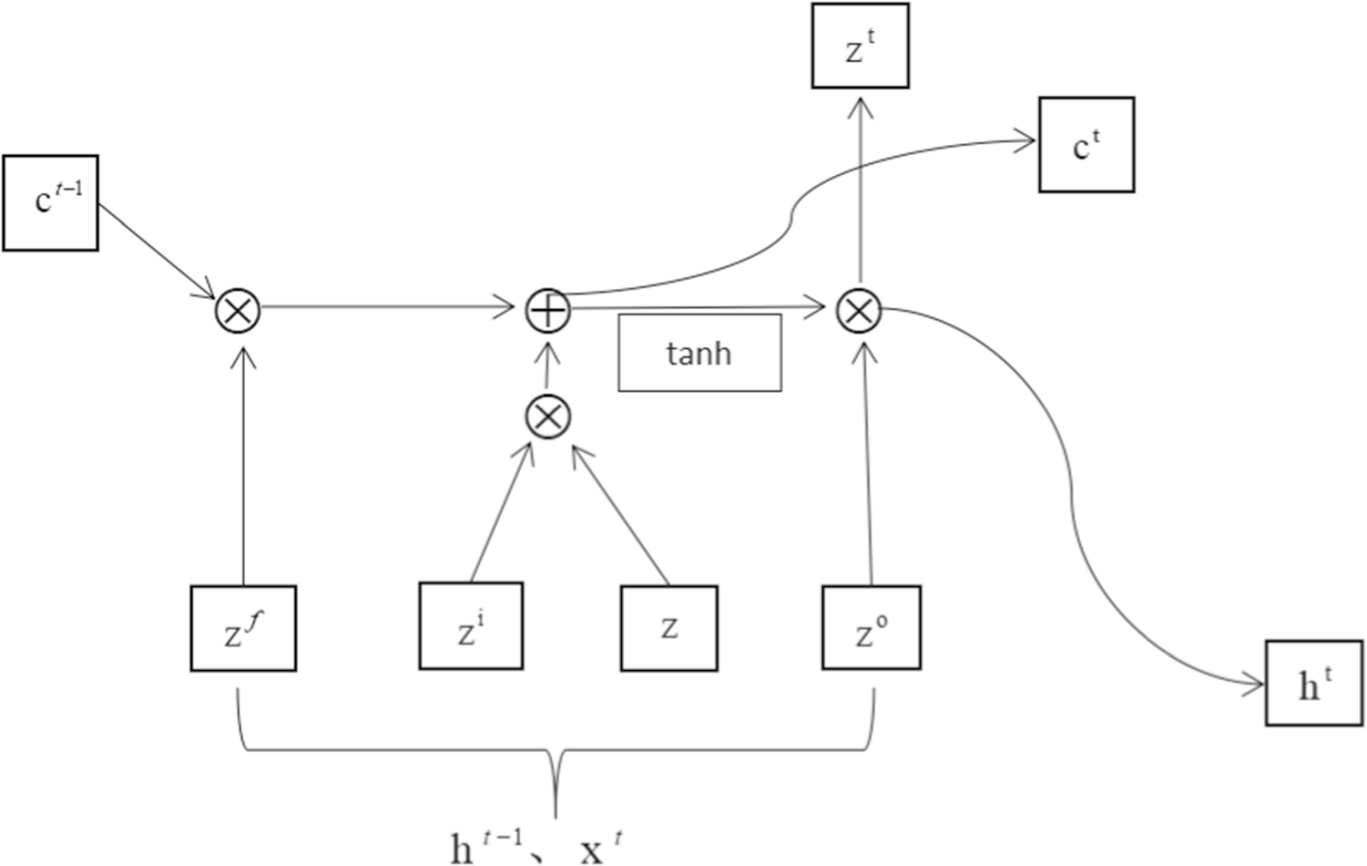
LSTM neurons diagram

Where RNN can be understood as having two inputs and two outputs, LSTM has three inputs and three outputs. $h^{t - 1}$ represents the status value of the previous hidden layer, and $x^{t} ,y^{t}$ represents the input data and the value derived from the model for output. The difference between the two in terms of the approximate model run steps is that $c^{t - 1}$ the value of the previous period of control short-term memory is added to LSTM. The following microscopic description of the differences in the neurons of LSTM is shown below (Fig. 2).

$Z^{{\text{f}}} ,Z^{i} ,Z^{{\text{o}}}$ represents the forgotten door, the input door, and the output door, respectively, and *Z* represents the value of the previous hidden layer state obtained through the softmax function. The final output of its neurons is achieved in the following steps:

(1) Select useful information and update the current state value by forgetting the door, while $w_{f} ,b_{f}$ represents the matrix and bias of the corresponding weights, respectively. $\sigma$ is the softmax function.

$$Z^{f} = \sigma \left( {w_{f} \times [h^{t - 1} ,x^{t} ] + b_{f} } \right)$$

(2) The input data are controlled through the input layer, and the memory unit status values are updated. $w_{i} ,b_{i}$ represent the corresponding weight matrix and bias, respectively.

$$\begin{gathered} Z^{i} = \sigma \left( {w_{i} \times [h^{t - 1} ,x^{t} ] + b_{i} } \right) \hfill \\ Z = \tanh \left( {w_{z} \times [h^{t - 1} ,x^{t} ] + b_{z} } \right) \hfill \\ \end{gathered}$$

(3) Calculate the status value of memory unit ${\text{c}}^{t}$ at time t.

$$c^{t} = z^{f} \times c^{t - 1} + z^{i} \times z$$

(4) The state value of the next hidden layer and the output value of this stage, $h^{t} ,y^{t}$, are calculated from the output layer.

$$\begin{gathered} z_{o} = \sigma \left( {w_{o} [h^{t - 1} ,x^{t} ] + b_{o} } \right) \hfill \\ h^{t} = z^{o} \times \tanh \left( {c^{t} } \right) \hfill \\ y^{t} = \sigma \left( {w_{y} h^{t} } \right) \hfill \\ \end{gathered}$$

Based on the abovementioned specific characteristics of LSTM and prediction steps, LSTM is different in that it increases the input threshold and forgets the threshold and output threshold so that the weight of the self-cycle is changed so that in the case of fixed model parameters, the integration scale at different times can be dynamically changed, thus avoiding the gradient disappearance or gradient expansion problem. Based on LSTM’s excellent performance in the time series model, this paper selects it as the benchmark forecasting model and combines several factor variables to forecast the exchange rates of JPY/USD, GBP/USD and CNY/USD and then shows its good prediction level by comparison with the traditional machine learning model random forest and the common multifactor boosting prediction method.

### Random Forest

**Fig. 3:**
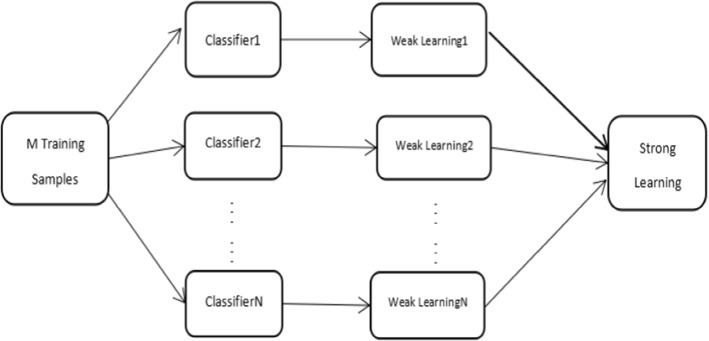
Integrated learning bagging

Random forest belongs to the category of integrated learning, which combines multiple weak supervision models to obtain a strong supervision model. Even if one of the weak models produces false predictions, other weak models can correct the errors. Bagging is characterized by no dependencies between weak learners, which can be used for parallel fitting. The following illustration shows a schematic for integrated learning bagging (Fig. 3).

Bagging takes a random, put-back sample, which randomly takes a fixed number of samples from inside the training set but puts the sample back after each sample is taken, meaning that previously collected samples may continue to be collected after they are put back. T weak learners are trained by the T sampling set, and then strong learners are generated by combining strategies, wherein the combined strategy of classification problems can be voted on: “minority obeys majority”, selecting the category with the largest number of votes as the final prediction, and the combined strategy of the regression problem is using the mean method: the arithmetic average of the results is used as the final prediction result.

### AdaBoost

The AdaBoost algorithm involves using very short (one-level) decision trees as weak learners that are added sequentially to the ensemble. Each subsequent model attempts to correct the predictions made by the model before it is in the sequence. This is achieved by weighing the training dataset to put more focus on training examples on which prior models made prediction errors. The steps for using the AdaBoost algorithm to predict the exchange rates are listed below:

(1) Initialize the weight distribution of the training data. Each training sample is initially given the same weight $\frac{1}{N}$_._

$$D_{1} = \left( {w_{11} ,w_{12} ,...,w_{1i} ,...,w_{1N} } \right),w_{1i} = \frac{1}{N},i = 1,2,...,N$$

(2) Multiple iterations are performed, with *m* = 1,2,…, *M* representing the number of iterations. Learn from the training dataset of *D*_*m*_ with weight distribution to obtain the basic classifier.

$$G_{m} (x):\chi \to \{ - 1, + 1\}$$

Calculate the classification error rate of *G*_*m*_ (*x*) on the training dataset.

$$e_{m}^{{}} = p(G_{m} (x_{i} ) \ne y_{i} ) = \sum\limits_{i = 1}^{N} {w_{mi} I(G_{m} (x_{i} ) \ne y)}$$

Calculate the coefficient of *G*_*m*_(*x*). $\alpha_{m}$ indicates how important *G*_*m*_(*x*) is in the final classifier.

$$\alpha_{m} = \frac{1}{2}\log \frac{{1 - e_{m} }}{{e_{m} }}$$

Update the weight distribution of the training dataset for the next round of iterations.

$$\begin{gathered} D_{m + 1} = \left( {w_{m + 1,1} ,w_{m + 1,2} ,...,w_{m + 1,i} ,...,w_{m + 1,N} } \right) \hfill \\ w_{m + 1,i} = \frac{{w_{mi} }}{{Z_{m} }}\exp \left( { - \alpha_{m} y_{i} G_{m} (x_{i} )} \right),i = 1,2,...,N \hfill \\ \end{gathered}$$

$Z_{m}$ is the normalization factor that makes $D_{m + 1}$ a probability distribution:

$$Z_{m} = \sum\limits_{i = 1}^{N} {w_{mi} \exp ( - \alpha_{m} y_{i} G_{m} (x_{i} ))}$$

Combine individual weak classifiers

$$f(x) = \sum\limits_{m = 1}^{M} {\alpha_{m} G_{m} (x)}$$

Thus, the final classifier is obtained as follows:

$$G(x) = sign(f(x)) = sign\left( {\sum\limits_{m = 1}^{M} {\alpha_{m} G_{m} (x)} } \right)$$

### Statistical Models

#### VAR Model

The VAR model we developed for exchange rate forecasting includes newly confirmed COVID-19 cases, exchange rate returns, market sentiment indicators, the market volatility index (VIX), and the spread between two benchmark interest rates. Vector autoregressive models are often used to predict interrelationship time series. In VAR models, there are no exogenous variables, and all variables in the model are explained by their own lagged terms and the lagged terms of other endogenous variables and random errors. Three-time series {${y}_{1t}$, ${y}_{2t}$, ${y}_{3t}$} are assumed to be the explanatory variables of the three regression equations, and the explanatory variables are the p-order lagged values of the two variables, constituting a ternary VAR(p) system.

$${y}_{1t}=\,{\beta }_{10}+{\beta }_{11}{y}_{1,t-1}+...+{\beta }_{p}{y}_{1t-p}+{\gamma }_{11}{y}_{2t-1}+...+{\gamma }_{1p}{y}_{2t-p}+...+{\alpha }_{11}{y}_{3t-1}+...{\alpha }_{1p}{y}_{3t-p}+{\epsilon }_{1t}$$

$${y}_{2t}=\,{\beta }_{20}+{\beta }_{21}{y}_{1,t-1}+...+{\beta }_{2p}{y}_{1t-p}+{\gamma }_{21}{y}_{2t-1}+...+{\gamma }_{2p}{y}_{2t-p}+...+{\alpha }_{21}{y}_{3t-1}+...{\alpha }_{2p}{y}_{3t-p}+{\epsilon }_{2t}$$

$${y}_{3t}=\,{\beta }_{30}+{\beta }_{31}{y}_{1,t-1}+...+{\beta }_{3p}{y}_{1t-p}+{\gamma }_{31}{y}_{2t-1}+...+{\gamma }_{3p}{y}_{2t-p}+...+{\alpha }_{31}{y}_{3t-1}+...{\alpha }_{3p}{y}_{3t-p}+{\epsilon }_{3t}$$

#### ARMA and GARCH Models

The autoregression moving average (ARMA) model is based on consolidating between the autoregressive model and the moving average model. The $AR(p)$ model is expressed as follows:

$$y_{t} = \alpha + \sum\limits_{i = 1}^{p} {\delta_{i} y_{t - i} + \varepsilon_{t} }$$

where $y_{t}$ is the actual value at time $t$, $\varepsilon_{t}$ is the random error at time $t$, $\delta_{i} \left( {i = 1,2, \ldots ,p} \right)$ are the autoregression parameters and $\alpha$ is a constant. The $MA(q)$ process is expressed as follows:

$$y_{t} = \alpha + \sum\limits_{j = 1}^{q} {\eta_{j} \varepsilon_{t - j} + \varepsilon_{t} }$$

where $\alpha$ is a constant, $\eta_{j} \left( {j = 1,2, \ldots ,q} \right)$ are the moving average parameters, and $q$ is the order of the model. $ARMA(p,q)$ model can then be formulated as follows:

$$y_{t} = \alpha + \sum\limits_{i = 1}^{p} {\delta_{i} y_{t - i} - \sum\limits_{j = 1}^{q} {\eta_{j} \varepsilon_{t - j} + \varepsilon_{t} } }$$

The $ARMA(p,q)$ model is widely used to analyze one-dimensional time series and variance constants. The value of the observations in the time series year can be expressed as a linear combination of the previous p-item observations and q-term random errors.

The autoregressive conditional heteroskedasticity (ARCH) model is a statistical technique used to examine and forecast conditional variances. According to the ARCH model, the variance of the time series is not constant and can be expressed as:

$$\sigma_{t}^{2} = \alpha + \sum\limits_{i = 1}^{q} {\theta_{i} \varepsilon_{t - i}^{2} }$$

where $\sigma_{t}^{2}$ is the conditional variance of random error $\varepsilon_{t}$ and $\theta_{i} \left( {i = 1,2, \ldots ,q} \right)$ is the parameter. The GARCH model seeks to simulate the path of financial time series through the statistical process proposed by Bollerslev [5]. It is defined as an equation of volatility and can be shown as:

$$\sigma_{t}^{2} = \alpha + \sum\limits_{i = 1}^{q} {\theta_{i} \varepsilon_{t - i}^{2} } + \sum\limits_{j = 1}^{p} {\beta_{j} \sigma_{t - j}^{2} }$$

## Data and Empirical Results

In this section, we first define the dependent, independent and control variables. The dependent variable is the return of the bilateral exchange rate of USD/JPY, GBP/USD, and USD/CNY. The independent variable is the variation in newly confirmed COVID-19 cases. Control variables include the benchmark interest rate spread between two countries, investors’ sentiment and the panic index obtained based on the S&P 500. The measurement of these variables is introduced. We then analyze the impact of newly confirmed COVID-19 cases on exchange rate dynamics using the ordinary least squares (OLS) method.

### Data and Variables

The number of newly confirmed COVID-19 cases in the selected countries, the benchmark interest rate spread between the two countries, investor sentiment and the panic index obtained based on the S&P 500 are selected to examine the impact on foreign exchange movement. The data are obtained from January 23rd, 2020 to September 14th, 2021. The study focuses on the effect of investor sentiment fluctuations due to the epidemic and the corresponding impact on the bilateral exchange rate.

The summary statistics of bilateral exchange rates, natural log of new COVID-19 confirmed cases, benchmark interest rates and market sentiment indicators are reported in Table 1.

**Table 1:** Summary statistics

Variable	Obs	Mean	Std.Dev	Min	Max
Panel A: Exchange rates					
USD/JPY	419	107.294	2.249	102.52	111.56
GBP/USD	419	1.327	0.062	1.149	1.421
USD/CNY	419	6.71	0.262	6.361	7.176
Panel B: COVID-19 confirmed cases (natural log)					
COVID2 (US)	408	10.551	1.776	0	12.754
COVID3 (JP)	404	6.805081	1.838	1.099	10.499
COVID4 (UK)	408	8.397	2.040	0	11.513
COVID1 (CN)	418	3.918	1.296	0	9.626
Panel C: Benchmark interest rates					
Fed Funds Rate (FFR)	419	0.169	0.339	0.04	1.59
Overnight Rate of Japan (ORJ)	419	− 0.102	0.407	− 3.90	− 0.01
LIBOR	419	0.085	0.147	0.032	0.686
SHIBOR	419	1.754	0.476	0.602	3.282
Panel D: Market sentiment					
CBOE Market Volatility Index (VIX)	419	26.194	10.958	13.68	82.69
Sentiment	419	− 0.319	0.212	− 1	1

#### Exchange Rate Measure

The exchange rate data used in our analysis were the exchange rates of the British pound (GBP), the Japanese yen (JPY), and the Chinese Yuan (CNY) against the US dollar (USD). We follow Panopoulou and Souropani’s [38] method to compute the exchange rate return:

$${Y}_{i,t}=\mathrm{ln}{S}_{i,t}-\mathrm{ln}{S}_{i,t-1}$$

where ${S}_{i,t}$ and ${S}_{i,t-1}$ denote the closing price of the bilateral exchange rate at times $t$ and $t-1$, respectively.

**Fig. 4:**
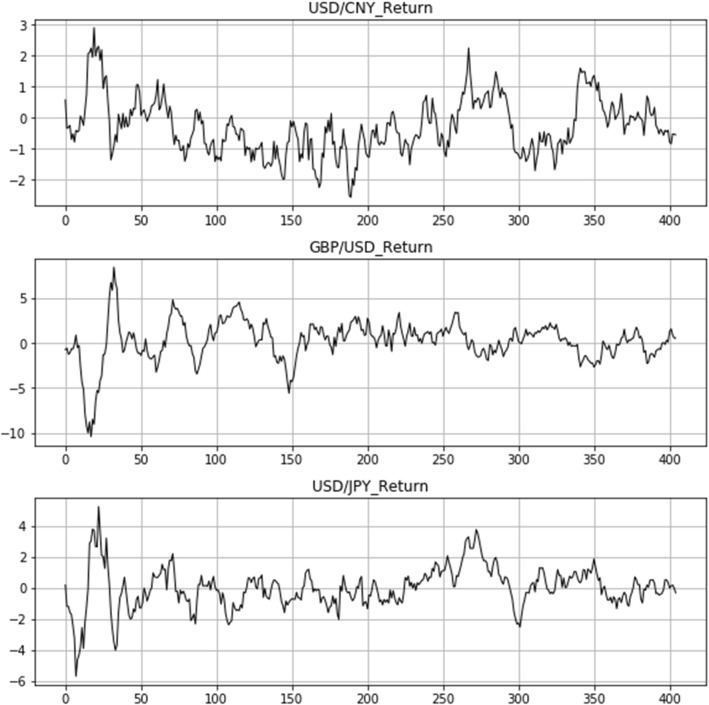
Exchange rate return

The computed exchange rate returns are demonstrated in Fig. 4. A rise in the USD/CNY yield represents the depreciation of CNY and the appreciation of USD. A rise in GBP/USD yield represents the appreciation of GBP and the depreciation of USD. A rise in the USD/JPY yield represents the depreciation of the JPY and the appreciation of the USD.

From the start of the outbreak until the end of the epidemic, Fig. 4 displays the 15-day rolling yield trend of the three exchange rates. In the first period (Day 0 to Day 50, the first wave of the pandemic), we have seen huge swings in exchange rate yields for the three nations as a result of the abrupt outbreak, leading to the conception that the panic about China’s epidemic is the reason for the CNY’s quick depreciation. The second period (Day 51 to Day 250, the second wave of the pandemic) has seen the stabilization of the return of three bilateral exchange rates, as the COVID19 vaccine has been used globally. In the third period (Day 251 afterward, the third wave of the pandemic), the return of bilateral exchanges demonstrated higher volatility than that in the second period as the Delta and Omicron variants came to light in the epidemic.

#### COVID19 Case Count

Different indicators, such as the number of newly confirmed infections, have been used to quantify the pandemic’s progression. Ding et al. [13] examine these indicators and conclude that the number of new infections is the most important factor influencing financial markets. As a result, it is used in some of the most important asset pricing studies looking at the influence of the COVID-19 epidemic on financial markets (e.g., [8, 35, 51]). Following these studies, we use the change in the number of confirmed cases as our major proxy for the shock of the pandemic.

We follow Ding et al. [13] and Zaremba et al. [51] to calculate the new COVID-19 confirmed cases:

$${COVID19}_{i,t}=\,\mathrm{ln}(1+{Cumulative\, Cases}_{i,t})-\mathrm{ln}(1+{Cumulative\, Cases}_{i,t-1})$$

where ${Cumulative\, Cases}_{i,t}$ and ${Cumulative\, Cases}_{i,t-1}$ represent the cumulative number of newly confirmed cases in country $i$ as of a 15-day rolling period ending at time $t$ and $t-1$, respectively. Thus, ${COVID19}_{i,t}$ measures the growth rate of newly confirmed cases over a 15-day rolling period in country $i$.

**Fig. 5:**
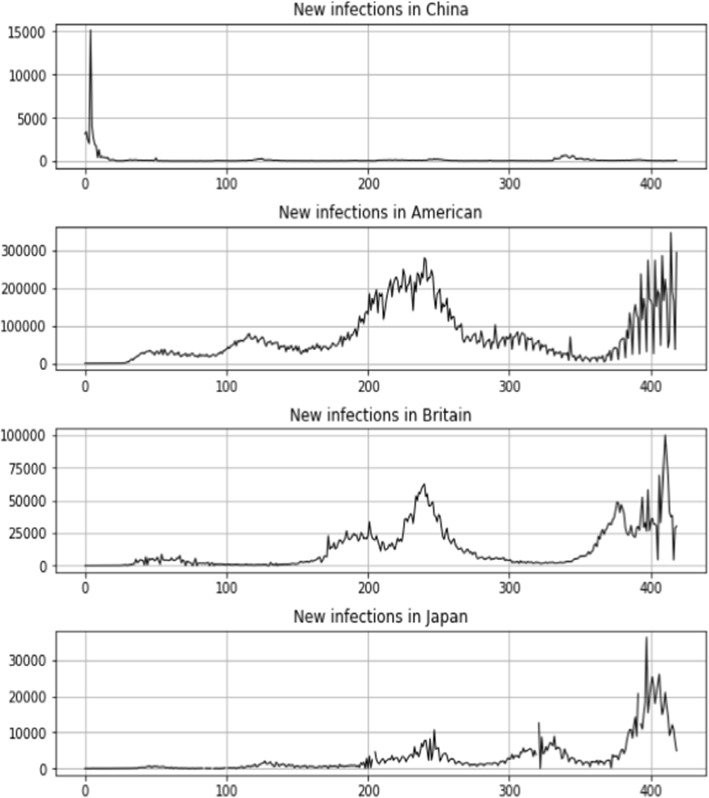
The number of newly confirmed COVID-19 cases in four countries

Figure 5 depicts the trend in the number of newly confirmed COVID-19 cases in four nations, as stated above. At the start of the epidemic, China had a much larger number of newly confirmed cases than the other nations. Before entering the stabilization stage (the second wave), the epidemic’s severe phase lasted approximately 50 days. With lockdown and social distancing policies being implemented and vaccines being used globally, the number of newly confirmed COVID-19 cases has been controlled in most countries. However, with the development of the Delta and Omicron variants and the gradual relaxation of lockdown controls in many countries except China, there has been a rapid increase in newly confirmed cases in the US, UK, and Japan.

#### Control Variables

We included in our analysis the benchmark interest rates of these countries as control variables. Different interrelationships between exchange rates and spreads have been obtained in the relevant literature and vary regarding the country and the duration of the spreads. Therefore, in the present study, the spread between two benchmark interest rates (IRM) is chosen as a control variable. Data on the Shanghai interbank offered rate (SHIBOR), London interbank offered rate (LIBOR), U.S. federal fund benchmark rate (FFR) and Japanese benchmark rate (ORJ) are obtained from the central bank’s website and reported in Table 1.

There is substantial evidence in the empirical literature on how investor sentiment influences capital markets such as the foreign exchange market. For instance, Wang et al. [46] examine the impact of investor sentiment on future stock market return rates. Shahzad et al. [44] investigate the relationship between investor sentiment and exchange rate return dependence. We study whether investor sentiment is one of the elements that impact foreign exchange in this research using the measure of market sentiment indicator.

Market sentiment indicators are constructed using text information obtained from major news websites and financial blogs and processed using the natural language processing (NLP) emotional procedure. The market sentiment indicator equals 1 if the sentiment is positive, indicating that the market is optimistic, 0 if the sentiment is neutral, and − 1 if the sentiment is negative. The sentiment index is then calculated as follows:

$$\mathrm{Sentiment}=\frac{\mathrm{postive}-\mathrm{negtive}}{\mathrm{postive}+\mathrm{negtive}}$$

The market volatility index (VIX) is often considered a barometer of the overall market, and many studies have used VIX information to predict energy volatility, stock market volatility or exchange rate volatility with better results.

### Empirical Results

The multivariate regression including the COVID-19 and market sentiment variables is expressed as follows:

$${\gamma }_{1}={\beta }_{1}+{\beta }_{2}{COVID19}_{1}+{\beta }_{3}{COVID19}_{2}+{\beta }_{4}Sentiment+{\beta }_{5}lnVIX+{\beta }_{6}{IRM}_{1}+{\varepsilon }_{1}$$

$${\gamma }_{2}={\beta }_{1}+{\beta }_{2}{COVID19}_{3}+{\beta }_{3}{COVID19}_{2}+{\beta }_{4}lnVIX{+{\beta }_{5}{IRM}_{2}+\varepsilon }_{2}$$

$${\gamma }_{3}={\beta }_{1}+{\beta }_{2}{COVID19}_{4}+{\beta }_{3}{COVID19}_{2}+{\beta }_{4}lnVIX{+{\beta }_{5}{IRM}_{3}+\varepsilon }_{3}$$

The empirical results of multivariate regression are reported in Table 2. It is obvious that the variation in newly confirmed COVID-19 cases has a significant effect in explaining the bilateral exchange rate movement of USD/CNY, USD/JPY, and GBP/USD.

**Table 2:** Multivariate regression results

	USD/CNY	USD/JPY	GBP/USD
COVID19-CN	− 0.0127** (0.00578)		
COVID19-US	0.00406*** (0.000626)	0.00465*** (0.00142)	0.00866*** (0.00156)
L.COVID19-JP		− 0.00654** (0.00315)	
COVID19-UK			− 0.00309* (0.00182)
lnVIX	− 0.00607*** (0.00196)	− 0.00680** (0.00264)	− 0.00998*** (0.00208)
Sentiment	0.0143*** (0.00536)		
US-CN IRM	− 0.000443 (0.00131)		
US-UK IRM			0.0181*** (0.00377)
US-JP IRM		− 0.00423 (0.00628)	
_cons	0.0186** (0.00724)	0.0233*** (0.00821)	0.0273*** (0.00657)

Standard errors in parentheses

*p < 0.10, **p < 0.05, ***p < 0.01

The market sentiment indicator significantly affects the movement of USD/CNY, while the market volatility index demonstrates a significant impact on the dynamics of the three bilateral exchange rates. Nevertheless, the impact of the spread of benchmark interest rates on the movement of exchange rates is not significant except for GBP/USD.

These results support the two basic hypotheses proposed in this paper. First, the variation in newly confirmed COVID-19 cases has played an influential role in determining the exchange rate movement. Second, both the VIX index and market sentiment have a significant impact on exchange rate dynamics.

### Exchange Rate Prediction Using Deep Learning and Statistical Models

In this section, we compare the performance of exchange rate prediction using various deep learning and statistical models. Three loss functions are selected as the evaluation criteria for the accuracy of various model predictions with reference to the empirical results. As reported in Table 3, ${L}_{1}$ is the root mean square error (RMSE), ${L}_{2}$ is the mean absolute error (MAE), and ${L}_{3}$ is the median absolute error (MedAE). The forecasting performance is better when the indicator’s value is smaller.

**Table 3:** Evaluating indicators

Evaluating indicator	Formula
RMSE	${L}_{1}=\sqrt{\frac{1}{n}\sum_{1}^{n}{y}_{t}-{\widehat{y}}_{t}}$
MAE	${L}_{2}=\sqrt{\frac{1}{n}\left|{y}_{t}-{\widehat{y}}_{t}\right|}$
MEDAE	${L_3} = median(\left| {{y_1} - {{\widehat y}_1},...,\left| {{y_t} - {{\widehat y}_t}} \right|} \right|)$

Table 4 shows the different loss function values of the 15-day rolling return of three bilateral exchange rates generated by five models. It is not difficult to conclude that the recurrent neural network (RNN) and long short-term memory (LSTM) models outperform the other deep learning models and VAR model in forecasting the bilateral exchange rate movement. Investor sentiment indicators play a vital role in enhancing the predictive power across different exchange rate forecasting models. RNN is the model with the best performance in forecasting USD/JPY and USD/CNY, while LSTM is the best in forecasting GBP/USD in the COVID-19 pandemic period.

**Table 4:** Measuring forecast performance using 15-day rolling return in the COVID19 pandemic period

	MAE	RMSE	MedAE
USD/JPY			
VAR	2.165	2.524	1.926
RF	1.674	2.061	1.443
AdaBoost	1.300	1.603	1.328
LSTM	1.470	1.850	1.300
RNN	1.229*	1.520*	1.099*
GBP/USD			
VAR	3.545	4.847	2.990
RF	1.026	1.251	1.000
AdaBoost	1.225	1.587	1.005
LSTM	0.612*	0.731*	0.561
RNN	0.634	0.785	0.556*
USD/CNY			
VAR	1.150	1.391	1.177
RF	0.447	0.593	0.313
AdaBoost	0.541	0.680	0.346
LSTM	0.330	0.435	0.296
RNN	0.321*	0.43*	0.278*

*Best forecasting performance in each category

**Fig. 6:**
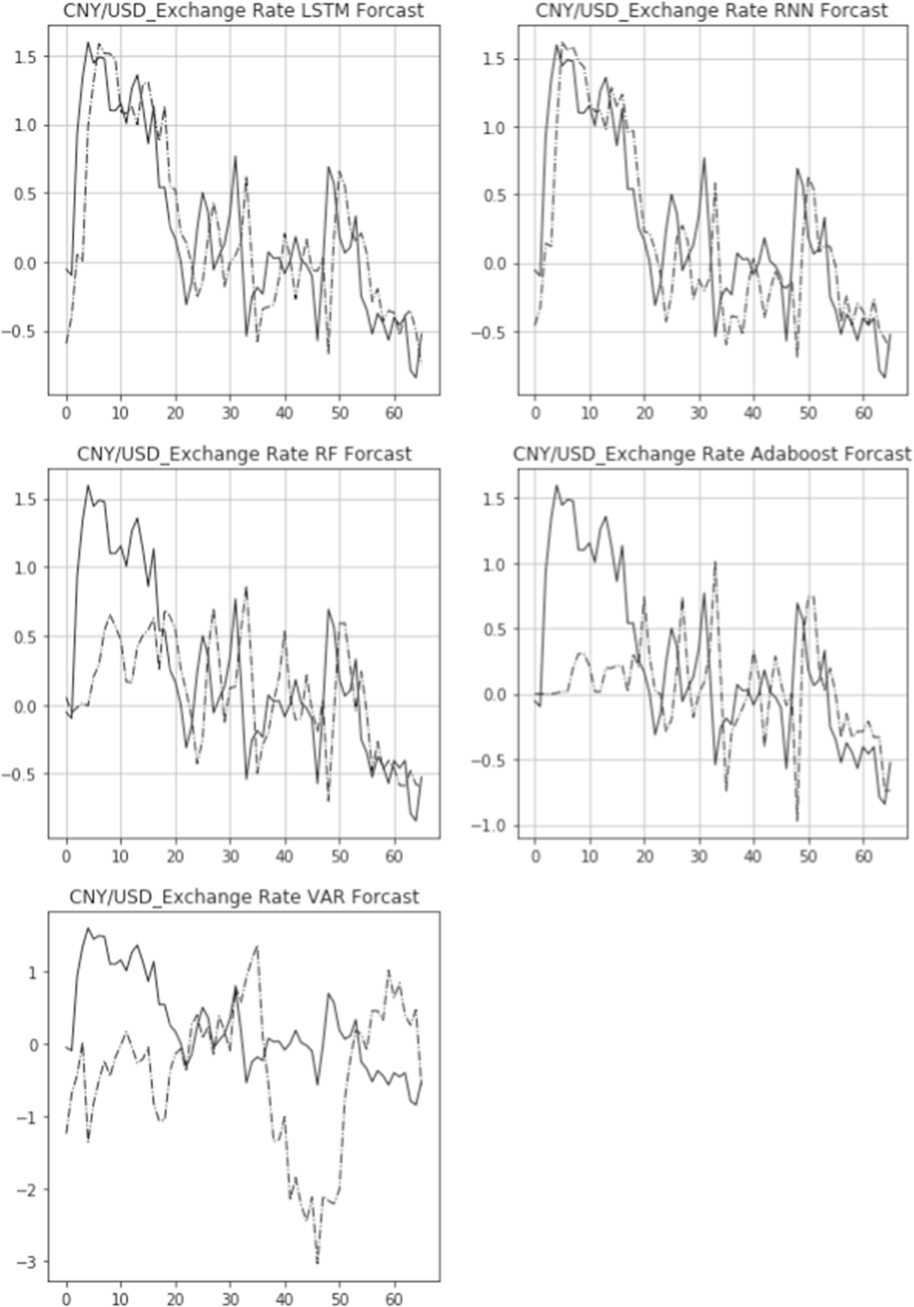
Comparison of actual and predicted exchange rate return (USD/CNY)

**Fig. 7:**
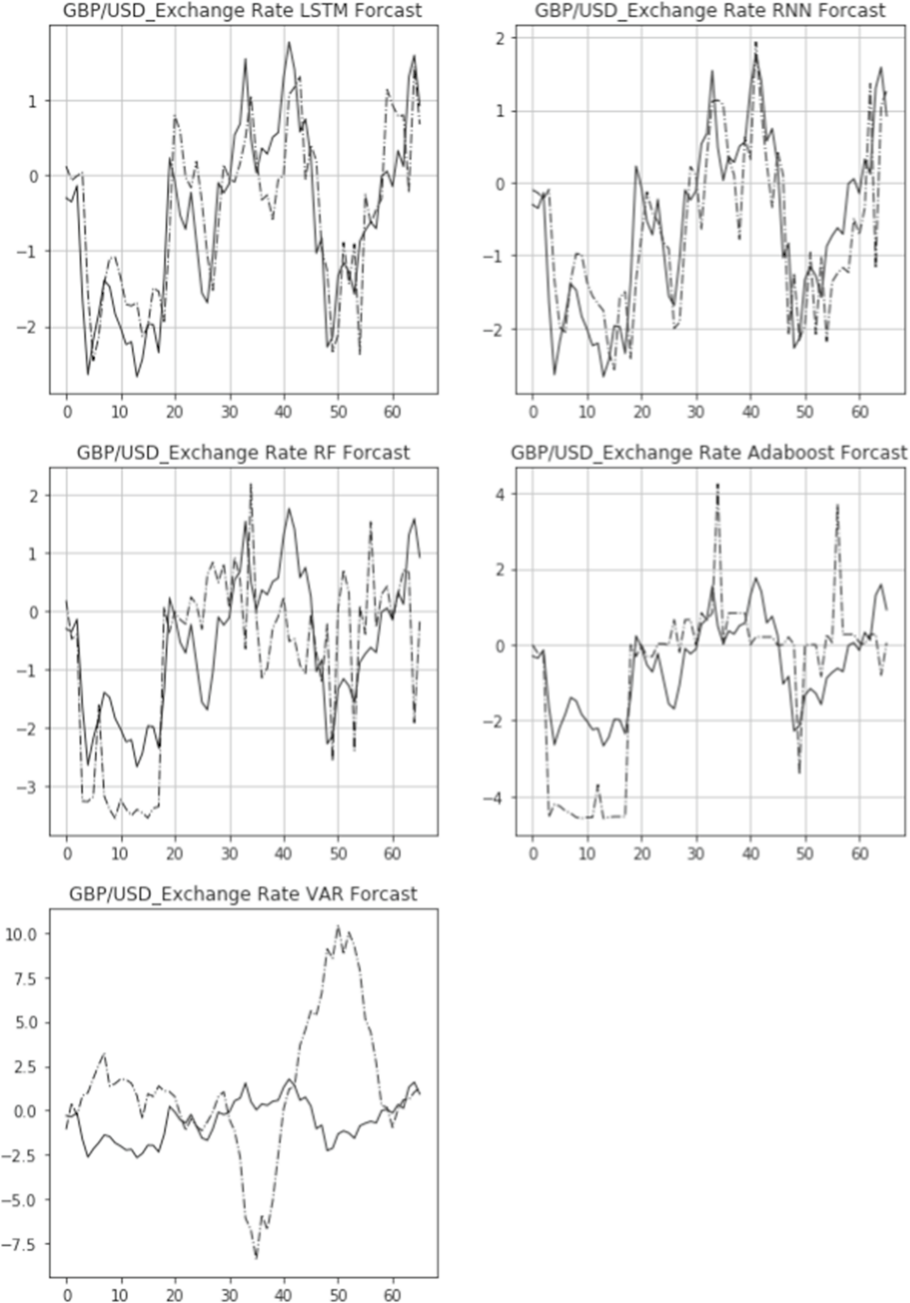
Comparison of actual and predicted exchange rate return (GBP/USD)

**Fig. 8:**
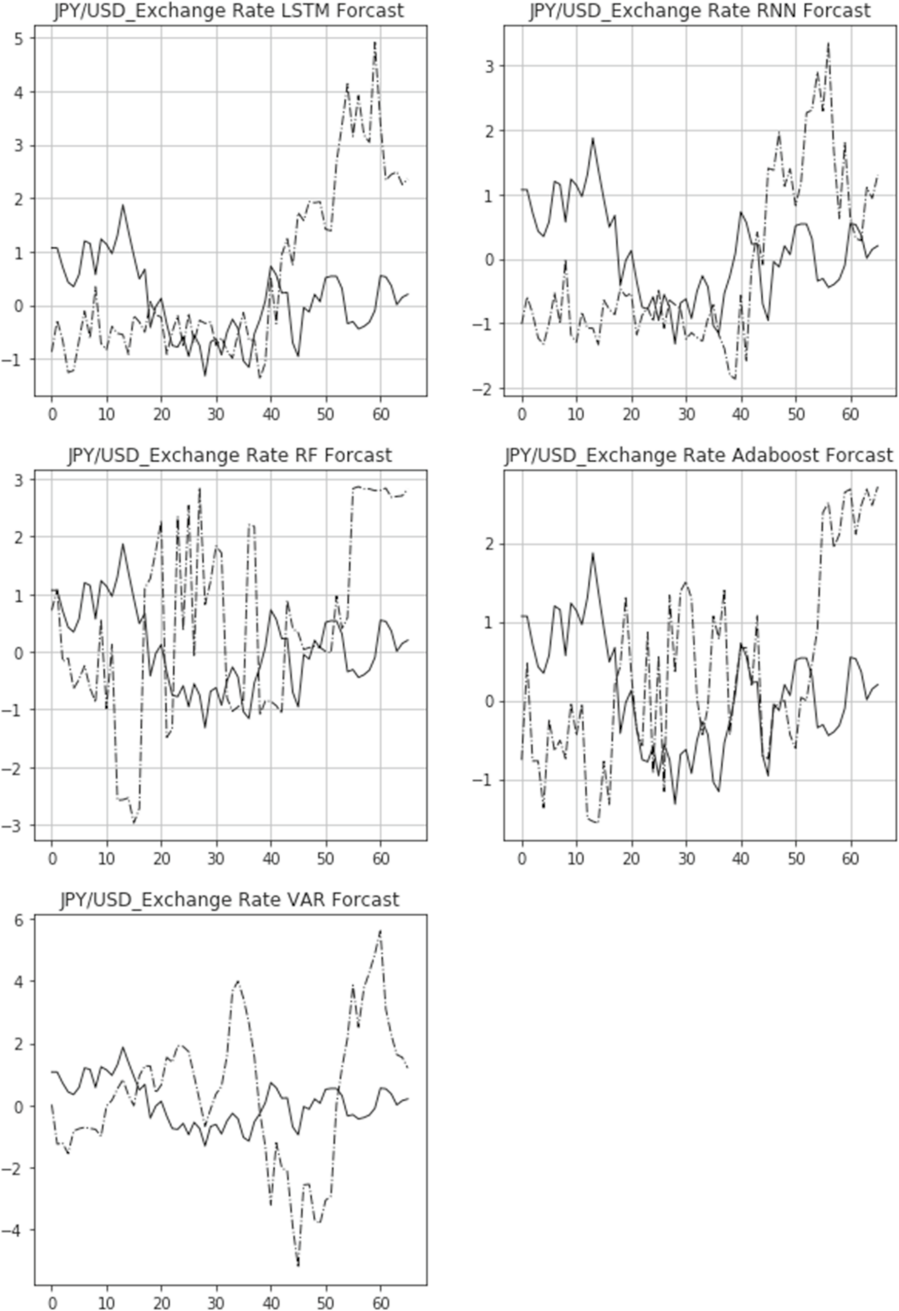
Comparison of actual and predicted exchange rate return (USD/JPY)

The actual 15-day rolling exchange rate return (solid line) and the predicted 15-day rolling return (dashed line) of three bilateral exchange rates, USD/CNY, GBP/USD, and USD/JPY, are shown in Figs. 6, 7, 8. From top to bottom are the images of the comparison of the LSTM, RNN, RF, Adaboost, and VAR models. The figures also demonstrate that the LSTM and RNN models fit relatively well with the predictions of different models for the exchange rate in the longitudinal direction.

### Robustness Check

To ascertain that the comparison results are robust, we replace the 15-day rolling exchange rate return with the daily exchange rate return to compare the performance of exchange rate prediction using different models. Table 5 reports the different loss function values of the daily return of three bilateral exchange rates generated by five models. In most scenarios, the results lend support to our findings that the RNN and LSTM models outperform the other deep learning models and VAR model in forecasting the bilateral exchange rate movement. Forecast performance using daily returns again supports that RNN is the model with the best performance in forecasting USD/JPY and USD/CNY, while LSTM is the best in forecasting GBP/USD in the COVID-19 pandemic period.

**Table 5:** Measuring forecast performance using daily return in the COVID-19 pandemic period

	MAE	RMSE	MedAE
USD/JPY			
VAR	0.535	0.735	0.347
RF	0.588	0.955	0.337*
AdaBoost	0.516	0.677	0.387
LSTM	0.500	0.642	0.386
RNN	0.440*	0.579*	0.392
GBP/USD			
VAR	0.729	1.022	0.538
RF	0.641	0.923	0.403*
AdaBoost	0.692	1.145	0.422
LSTM	0.548*	0.691*	0.416
RNN	0.593	0.750	0.472
USD/CNY			
VAR	0.304	0.442	0.206
RF	0.251	0.323	0.187
AdaBoost	0.239	0.341	0.178
LSTM	0.218	0.287	0.152*
RNN	0.215*	0.291*	0.167

*Best forecasting performance in each category

**Fig. 9:**
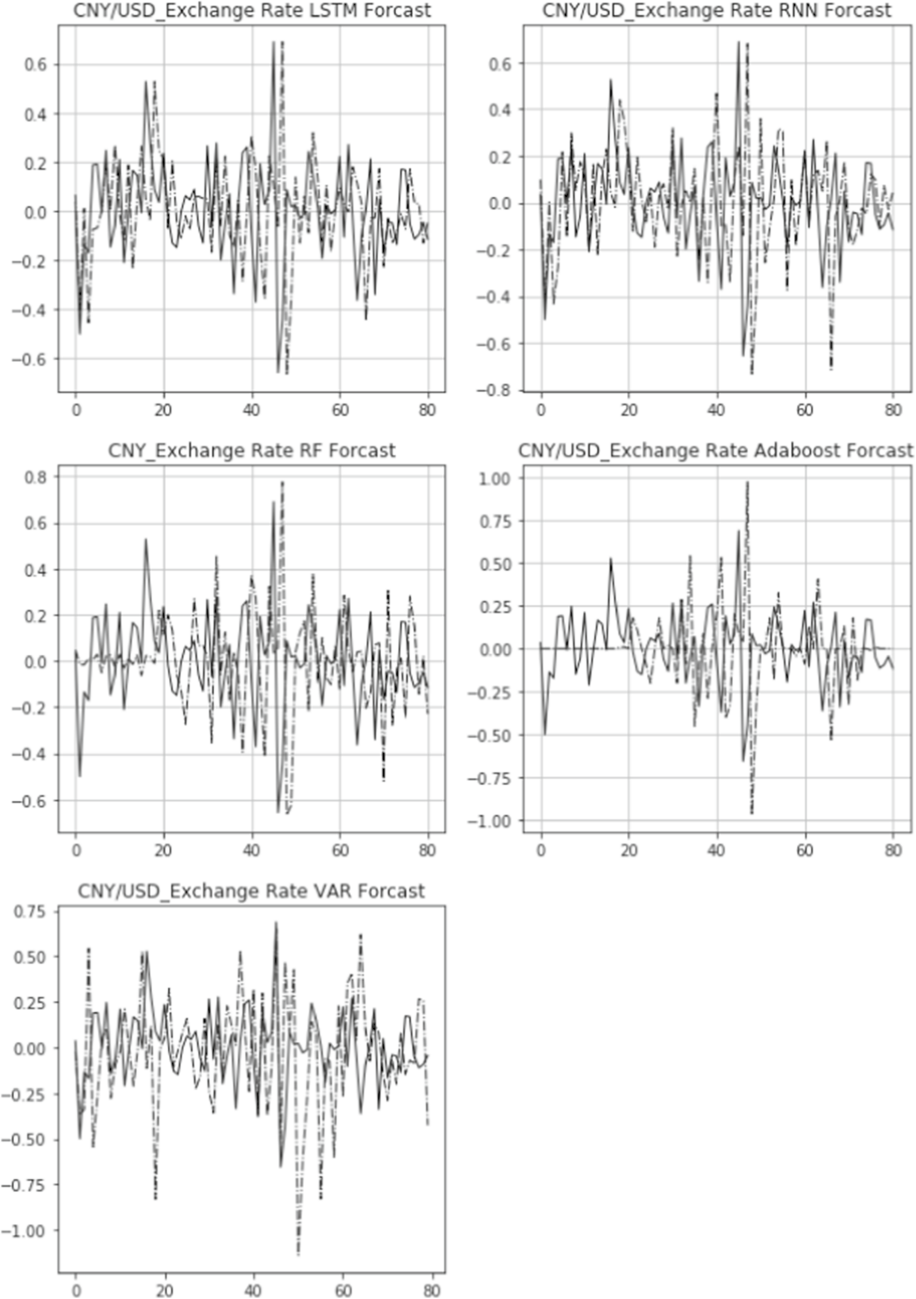
Robustness check: comparison of actual and predicted exchange rate return (USD/CNY)

**Fig. 10:**
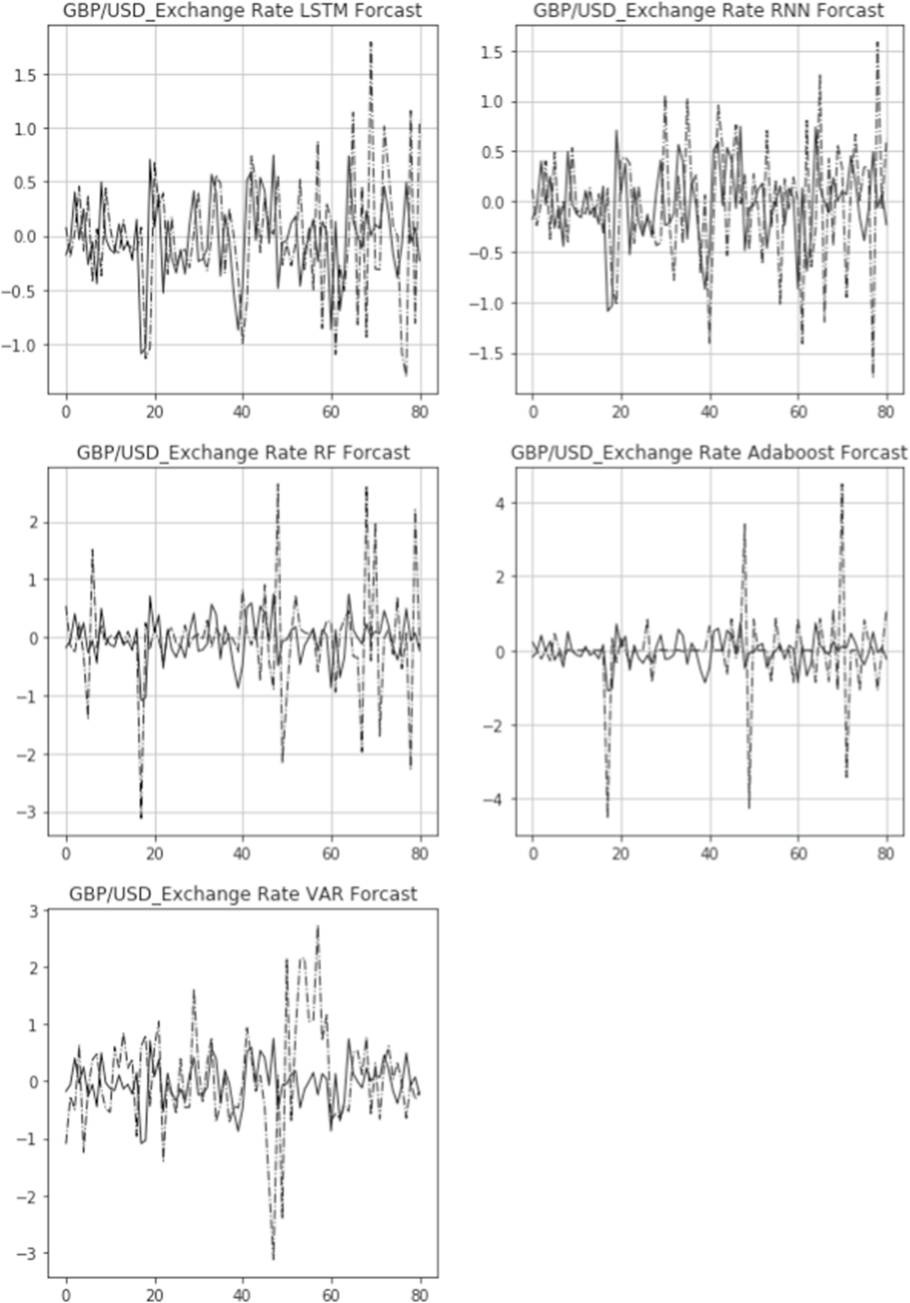
Robustness check: comparison of actual and predicted exchange rate return (GBP/USD)

**Fig. 11:**
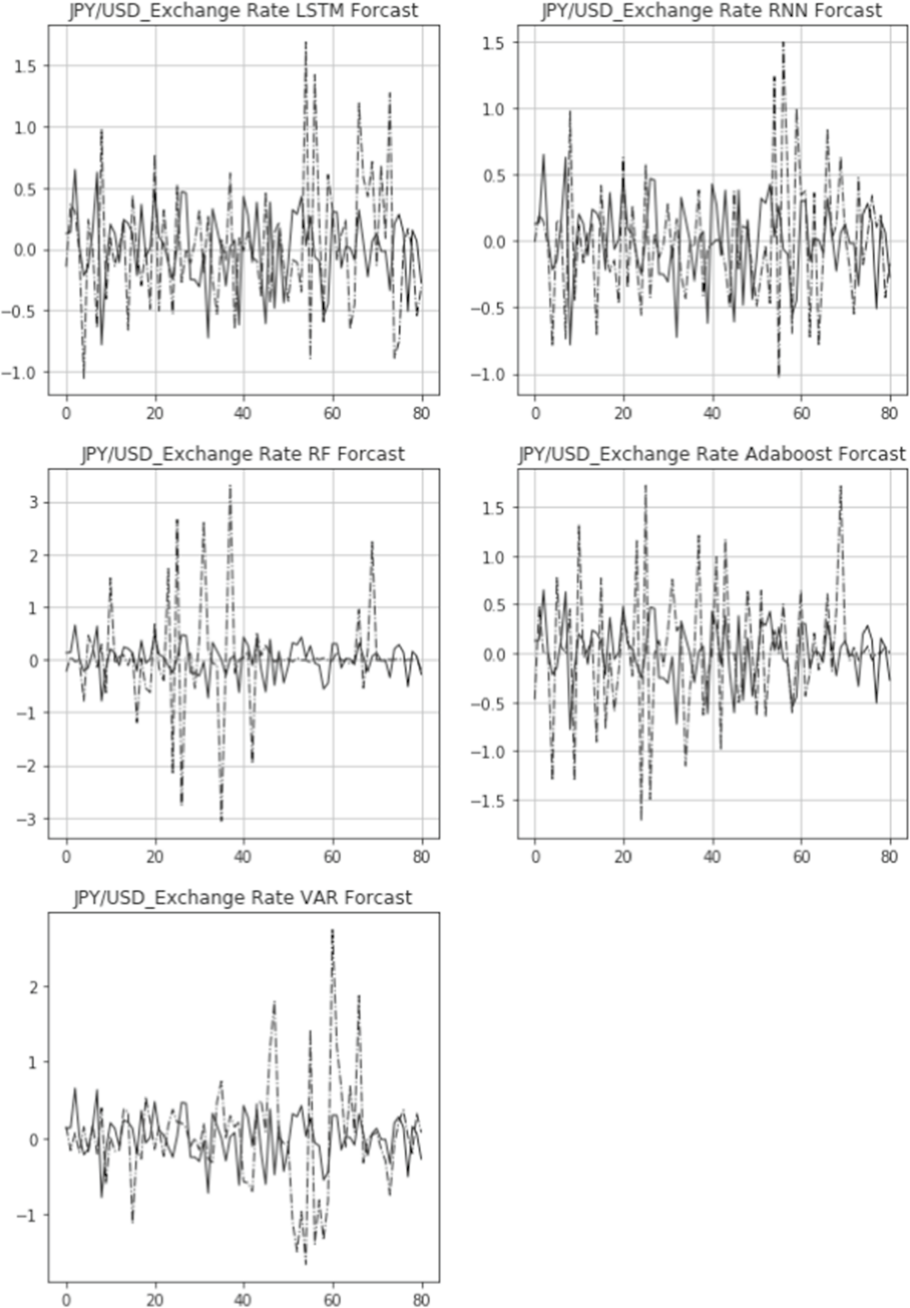
Robustness check: comparison of actual and predicted exchange rate return (USD/JPY)

Figures 9, 10, 11 display the comparison of the actual daily exchange rate return (solid line) and the predicted daily return (dashed line) of three bilateral exchange rates, USD/CNY, GBP/USD, and USD/JPY, using five models. It also shows that the LSTM and RNN models fit relatively well with the predictions of different models for the exchange rate dynamics.

## Further Analysis Using the Ensemble Learning Method and High-Frequency Data

In this section, we further examine the intraday prediction of the bilateral exchange rate. We first introduce the ensemble learning method and application of bagging and boosting. Then, a group of widely used technical indicators is selected and used in the integrated statistical and deep learning models. The prediction results based on GARCH + LSTM, GARCH + RNN, GARCH + Adaboost, GARCH + RF, GARCH + LightGBM, and GARCH + GBDT are compared with those based on GARCH and ARMA models.

### Ensemble Learning Method

Single deep learning models, such as ANN and SVR, are sensitive to parameters, and their performances are readily influenced by unique scenarios. As a result, the single model’s accuracy and stability must be enhanced [34]. In this case, the ensemble learning method [7] is recommended to improve the single model’s accuracy and stability by combining many base learners to produce the aggregate result [52].

In general, ensemble learning is a model that produces predictions based on several distinct models. Ensemble learning is more flexible (less bias) and less data-sensitive since it combines several distinct models (less variance). Bagging and boosting are the two most prevalent ensemble learning strategies. Bagging is the process of simultaneously training a number of models. Each model is trained on a portion of the data that is chosen at random. Boosting is the process of successively training several models. Each model learns from the preceding model’s errors.

Ensemble learning is proposed to combine many base learners to compensate for the inadequacies of the single model. Ensemble learning may provide a more stable and accurate outcome by establishing various base learners and merging the base learning results. There have been studies that have used ensemble learning to improve prediction performance in exchange rate forecasting.

To gather numerous training sets and construct base learners based on the training sets, Yu et al. [48] employed bagging and boosting. The experimental findings revealed that ensemble learning’s prediction impacts were often superior to those of a single model. The empirical analysis of four main daily exchange rate datasets indicated that the Adaboost ensemble learning strategy was optimal. Wu and Gao [47] used Adaboost to aggregate the findings of single predictors.

### GBDT and LightGBM

The application of bagging is found in random forests. Random forests are a parallel combination of decision trees. Each tree is trained on a random subset of the same data, and the results from all trees are averaged to find the classification. The application of boosting is found in gradient boosting decision trees (GBDTs).

Many weak learners were united in GBDT to produce one strong learner. Individual decision trees are the poor learners in this case. All of the trees are connected in succession, with each tree attempting to reduce the mistake of the one before it. Boosting algorithms are often difficult to train but extremely precise due to this sequential relationship. As the model improves, the weak learners are fitted in such a way that each new learner fits into the residuals of the preceding stage. The final model combines the results of each phase, resulting in a strong learner.

Light Gradient Boosting Machine (LightGBM) is a new GBDT technique presented by Ke et al. [25] that has been employed in a variety of data mining applications, including classification, regression, and ordering. The gradient-based one-side sampling and exclusive feature bundling approaches in the LightGBM algorithm are also innovative techniques.

### Selection of Technical Indicators and Model Parameters

Table 6 shows the set of widely used technical indicators suggested by Kara et al. [24] and Alonso-Monsalve et al. [2] for the feature extraction of high-frequency data in the present study. This group of indicators comprises momentum indicators, moving average convergence/divergence indicators, relative strength index indicators, and so on. Among them, the moving average is frequently used to build trading rules that generate buy and sell signals based on the relative behavior of indicators evaluated over different time periods.

**Table 6:** Selected technical indicators

Indicator	Formula
MACD	${MACD(n)}_{t-1}+2/n-1*({DF}_{t}-{MACD(n)}_{t-1})$
A/D	$\frac{{H}_{t}-{C}_{t-1}}{{H}_{t}-{L}_{t}}$
LWR	$\frac{{H}_{N}-{C}_{t}}{{H}_{n}-{L}_{n}}$
RSI	100$-\frac{100}{1+(\sum_{n=0}^{n-1}{up}_{t-i}/n)/(\sum_{n=0}^{n-1}{DW}_{t-i}/n)}$
SMA (5,10,20,30,60)	$\frac{{C}_{t}+{C}_{t-1}+...+{C}_{t-n+1}}{n}$
WMA (5,10,20,30,60)	$\frac{{n*C}_{t}+(n-1){C}_{t-1}+...+{C}_{t-n+1}}{n+(n-1)+...+1}$
SD	$\frac{\sum_{n=0}^{n-1}{K}_{t-i}}{n}$
SK	$\frac{{C}_{t}-{LL}_{t-n}}{{HH}_{t-n}-{LL}_{t-n}}$
CCI	$\frac{{M}_{t}-{SM}_{t}}{0.015{D}_{t}}$
Momentum	${C}_{t}$-${C}_{t-n}$

${C}_{t}:$ closing price; ${L}_{t}$: lowest price; ${H}_{t}$: highest price at time t; DF:${\mathrm{EMA}(12)}_{t}$-${\mathrm{EMA}(26)}_{t}$; EMA: exponential moving average; ${\mathrm{EMA}(\mathrm{K})}_{t}:{\mathrm{EMA}(\mathrm{K})}_{t-1}+\alpha *({C}_{t}-{\mathrm{EMA}(\mathrm{K})}_{t-1})$; $\mathrm{\alpha }$: smoothing factor: 2/1 + k; k:time period of k minute exponential moving average; ${LL}_{t}$ and ${HH}_{t}:$ mean lowest low and highest high in the last t minutes; ${M}_{t}$:${H}_{t}$+${L}_{t}$+${C}_{t}$/3; ${SM}_{t}$:$\sum_{i=1}^{n}{M}_{t-i+1}/n$; ${D}_{t}$:($\sum_{i=1}^{n}{|M}_{t-i+1}-{SM}_{t}|$)/n; ${up}_{t}$:upward price change;${DW}_{t}$:down price change at time t

The comparison of these lagging indicators exposes changes based on financial time series patterns. Momentum indicators track the pace of price changes by measuring price differences over relatively short time periods. These indicators are used by investors to determine the strength of a trend and are frequently used to forecast reversals and, as a result, establish trading signals.

To perform the prediction integrating the statistical models and deep learning algorithms, the original exchange rate data are first computed as a return and then calculated by the GARCH model as an input variable together with other factors into the two deep learning models (RNN and LSTM) and ensemble learning algorithms (Adaboost, RF, LightGBM, GBDT) to achieve the prediction results. The best forecasting results achieved by selecting parameters in the forecasting model are shown in Table 7.

**Table 7:** Model parameter selection

Model	Parameter
LSTM	units = 100/70, epochs = 150/100, batch_size = 300/120
RNN	units = 100/70, epochs = 150/100, batch_size = 300/120
GBDT	learning_rate = 0.05,n_estimators = 80,subsample = 0.7,max_depth = 8,min_samples_leaf = 8
Adaboost	n_estimators = 80,learning_rate = 0.05,random_state = 200
LightGBM	learning_rate = 0.05,max_depth = 5,min_data_in_leaf = 10,min_sum_hessian_in_leaf = 2.4,min_gain_to_split = 0.03,feature_fraction = 0.7,bagging_fraction = 0.8,bagging_freq = 10,lambda_l1 = 1.1,lambda_l2 = 0.3
RF	n_estimators = 20,max_depth = 20,min_samples_leaf = 20

### High-Frequency Data and Prediction Performance

In this section, high-frequency intraday data of USD/CNY, GBP/USD, and USD/JPY and the exchange rate return are presented in both the 30-min and 60-min intervals. The sample period is from January 2, 2009 to September 14, 2021.

**Fig. 12:**
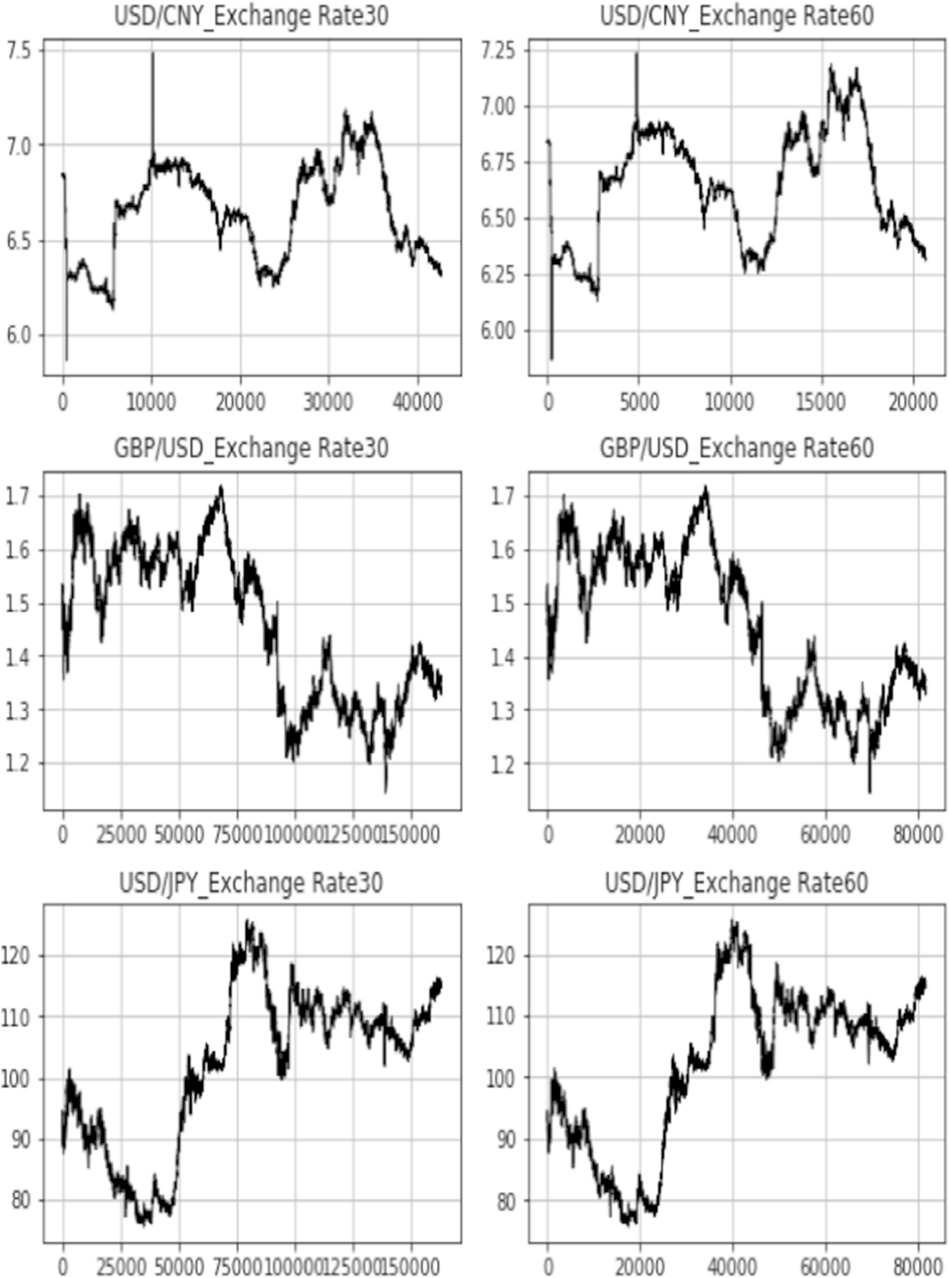
High-frequency bilateral exchange rate: 30-min and 60-min intraday data

**Fig. 13:**
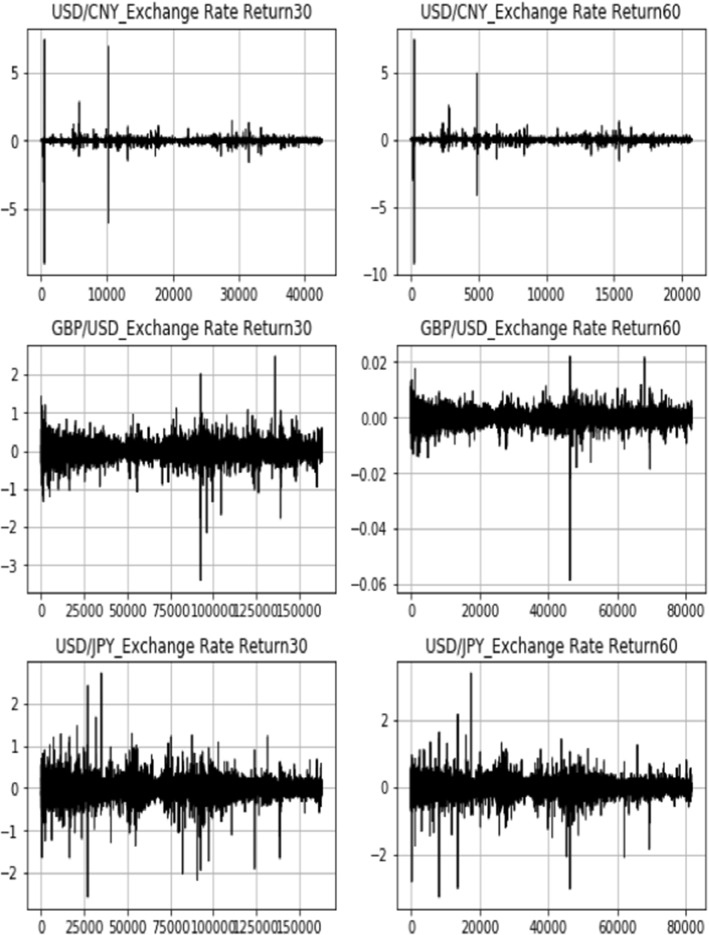
High-frequency bilateral exchange rate return: 30-min and 60-min intraday data

Figure 12 shows the intraday exchange rate data of USD/CNY, GBP/USD, and USD/JPY in 30- and 60-min intervals. Both the GBP and JPY display a depreciation pattern against the US dollar during the sample period. The CNY, nevertheless, followed a different path [18]. In contrast to the de facto fixed exchange rate system before the reform, the CNY exchange rate swings became more market-oriented with a broader scope, higher intensity, and more sensitivity to external variables after the exchange rate system reform in 2005. Furthermore, since the reform until the end of January 2014, the exchange rate of USD/CNY has been changing and growing, with an appreciation rate of over 25%. However, beginning in February 2014, the CNY began a trend of continual devaluation, declining by nearly 13.56% versus the US dollar by December 2016 (Fig. 13).

The results of prediction integrating the statistical models and deep learning algorithms using 30- and 60-min intraday data are reported in Table 8. The MAE, Medae, and RMSE values of GARCH + RF and GARCH + GBDT are substantially less than those of other ensemble models. In particular, GARCH + GBDT achieved the best performance in forecasting the intraday movement of USD/CNY and USD/JPY in both 30- and 60-min intervals and GBP/USD in 60-min intervals. GARCH + RF achieved the best performance in forecasting the intraday movement of GBP/USD in 30-min intervals.

**Table 8:** Measuring forecast performance using 30-min and 60-min intraday returns during 2009–2021

CNY_30mins	RMSE	MAE	MEDAE	CNY_60mins	RMSE	MAE	MEDAE
GARCH + LSTM	0.024	0.017	0.013	GARCH + LSTM	0.037	0.026	0.020
GARCH + RNN	0.018	0.013	0.010	GARCH + RNN	0.041	0.037	0.035
GARCH + Adaboost	0.039	0.023	0.014	GARCH + Adaboost	0.057	0.036	0.023
GARCH + RF	0.015	0.011	0.006	GARCH + RF	0.018	0.014	0.008
GARCH + LightGBM	0.024	0.014	0.007	GARCH + LightGBM	0.024	0.014	0.007*
GARCH + GBDT	0.011*	0.007*	0.004*	GARCH + GBDT	0.016*	0.010*	0.008
GARCH	0.134	0.032	0.015	GARCH	0.807	0.651	0.085
ARMA	0.097	0.029	0.014	ARMA	0.127	0.041	0.019

GBP_30mins	RMSE	MAE	MEDAE	GBP_60mins	RMSE	MAE	MEDAE
GARCH + LSTM	0.028	0.023	0.020	GARCH + LSTM	0.04	0.03	0.025
GARCH + RNN	0.030	0.021	0.018	GARCH + RNN	0.043	0.029	0.027
GARCH + Adaboost	0.033	0.022	0.015	GARCH + Adaboost	0.05	0.030	0.019
GARCH + RF	0.018*	0.013*	0.009*	GARCH + RF	0.041	0.023	0.016
GARCH + LightGBM	0.025	0.017	0.015	GARCH + LightGBM	0.046	0.026	0.017
GARCH + GBDT	0.020	0.140	0.009*	GARCH + GBDT	0.035*	0.022*	0.014*
GARCH	0.390	0.107	0.087	GARCH	0.206	0.168	0.148
ARMA	0.125	0.055	0.022	ARMA	0.175	0.107	0.065

JPY_30mins	RMSE	MAE	MEDAE	JPY_60mins	RMSE	MAE	MEDAE
GARCH + LSTM	0.021	0.016	0.013	GARCH + LSTM	0.039	0.031	0.023
GARCH + RNN	0.022	0.044	0.016	GARCH + RNN	0.040	0.033	0.030
GARCH + Adaboost	0.020	0.014	0.010	GARCH + Adaboost	0.041	0.037	0.035
GARCH + RF	0.016	0.012	0.009	GARCH + RF	0.026	0.02	0.015
GARCH + LightGBM	0.015	0.011	0.008	GARCH + LightGBM	0.028	0.023	0.020
GARCH + GBDT	0.014*	0.010*	0.007*	GARCH + GBDT	0.020*	0.015*	0.012*
GARCH	0.206	0.169	0.074	GARCH	0.180	0.120	0.090
ARMA	0.122	0.053	0.022	ARMA	0.176	0.130	0.102

*Best forecasting performance in each category

Generally, LightGBM, GBDT, Adaboost, LSTM, and RNN produce lower MAE, Medae, and RMSE values than the traditional statistical models, such as GARCH and ARMA, indicating that ensemble models can significantly improve the accuracy of exchange rate prediction and that the integrated learning models and deep learning models are more effective than traditional statistical models, implying that the former is better suited to forecasting intraday exchange rate movement than the latter.

The results of prediction integrating the statistical models and deep learning algorithms using daily data in the 2009–2021 period and the recent COVID-19 pandemic period are reported in Table 9. In many cases, the MAE, Medae, and RMSE values of GARCH + RF and GARCH + GBDT are less than those of other ensemble models. There are some exceptions. For instance, GARCH + GBDT and GARCH + AdaBoost show better prediction performance for the bilateral exchange rate of USD/JPY in the COVID-19 period.

**Table 9:** Measuring forecast performance using daily returns during 2009–2021 and COVID-19 pandemic period

CNY	RMSE	MAE	MEDAE	CNY_Cov_period	RMSE	MAE	MEDAE
GARCH + LSTM	0.211	0.158	0.118	GARCH + LSTM	0.232	0.172	0.119
GARCH + RNN	0.343	0.287	0.268	GARCH + RNN	0.227	0.166	0.117
GARCH + Adaboost	0.209	0.149	0.104	GARCH + Adaboost	0.225	0.165	0.126
GARCH + RF	0.206	0.147*	0.103	GARCH + RF	0.223	0.164	0.128
GARCH + LightGBM	0.205*	0.147*	0.104	GARCH + LightGBM	0.276	0.166	0.111*
GARCH + GBDT	0.205*	0.149	0.101*	GARCH + GBDT	0.200*	0.161*	0.122
GARCH	0.296	0.212	0.102	GARCH	0.396	0.334	0.307
ARMA	0.207	0.1	0.224	ARMA	0.394	0.305	0.238

GBP	RMSE	MAE	MEDAE	GBP_Cov_period	RMSE	MAE	MEDAE
GARCH + LSTM	0.507	0.386	0.307	GARCH + LSTM	0.062	0.06	0.055
GARCH + RNN	0.571	0.444	0.352	GARCH + RNN	0.037*	0.031*	0.028*
GARCH + Adaboost	0.512	0.392	0.307*	GARCH + Adaboost	0.048	0.045	0.04
GARCH + RF	0.505*	0.385*	0.309	GARCH + RF	0.04	0.037	0.036
GARCH + LightGBM	0.506	0.386	0.308	GARCH + LightGBM	0.044	0.042	0.043
GARCH + GBDT	0.517	0.394	0.315	GARCH + GBDT	0.055	0.054	0.035
GARCH	0.837	0.651	0.552	GARCH	0.779	0.759	0.780
ARMA	0.613	0.443	0.341	ARMA	0.978	0.922	0.963

JPY	RMSE	MAE	MEDAE	JPY_Cov_period	RMSE	MAE	MEDAE
GARCH + LSTM	0.449	0.354	0.299	GARCH + LSTM	0.305	0.236	0.19
GARCH + RNN	0.472	0.374	0.321	GARCH + RNN	0.318	0.239	0.184
GARCH + Adaboost	0.394	0.302	0.237	GARCH + Adaboost	0.306	0.228*	0.186
GARCH + RF	0.404	0.313	0.163*	GARCH + RF	0.306	0.246	0.182
GARCH + LightGBM	0.384*	0.290*	0.217	GARCH + LightGBM	0.305	0.238	0.184
GARCH + GBDT	0.4	0.305	0.248	GARCH + GBDT	0.302*	0.237	0.179*
GARCH	0.835	0.657	0.551	GARCH	0.598	0.455	0.383
ARMA	0.843	0.424	0.44	ARMA	0.434	0.295	0.21

*Best forecasting performance in each category

The comparison between the prediction results based on high-frequency data and daily data reveals that the larger the dataset is, the better the prediction results provided by the ensemble models integrating the statistical models and deep learning algorithms. In particular, the accuracy of prediction results based on 30-min intraday data is better than the prediction results based on 60-min intraday data.

Finally, the classic econometric model fails miserably in projecting huge data, with the accuracy gap narrowing only on a daily basis. Ensemble learning models are more promising in exchange rate forecasting than individual models because ensemble learning is better at coping with the nonlinear and nonstationary features of exchange rate time series than single models.

## Conclusion

This paper attempts to investigate the impact of the COVID-19 pandemic and market sentiment on the dynamics of USD/JPY, GBP/USD, and USD/CNY. We compose the market sentiment variable and incorporate the COVID-19 confirmed cases and sentiment variable into the traditional exchange rate model. We find that confirmed COVID-19 cases and sentiment variables in the US, Japan, UK, and China in the period of January 23rd, 2020 to September 14th, 2021 are significant in explaining the bilateral exchange rate movement. After comparing the performance of the VAR model and four deep learning models during the COVID-19 pandemic, we conclude that the RNN and LSTM models outperform the other deep learning models and VAR model in forecasting the bilateral exchange rate movement.

Further analysis using high-frequency intraday data and ensemble models shows that ensemble models significantly improve the accuracy of exchange rate prediction, as they are better at coping with the nonlinear and nonstationary features of exchange rate time series. The ensemble models integrating the statistical models and deep learning algorithms outperform the individual econometric model or individual deep learning model in exchange rate forecasting. In particular, GARCH + GBDT shows the best prediction performance for USD/CNY and USD/JPY using both 30- and 60-min data and for GBP/USD using 60-min data.

## Data Availability

The datasets generated during and/or analyzed during the current study are available from the corresponding author upon reasonable request.

## Abbreviations

RNN: Recurrent neural network
LSTM: Long short-term memory
VAR: Vector autoregressive
ECB: European Central Bank
FED: Federal Reserve Bank
ARMA: Autoregression moving average
ARIMA: Autoregressive integrated moving average
ARCH: Autoregressive conditional heteroskedasticity
GARCH: Generalized autoregressive conditional heteroskedasticity
ANN: Artificial neural network
SVM: Support vector machine
SVR: Support vector regression
BPNN: Back propagation neural network
RF: Random forest
Adaboost: Adaptive boosting
VIX: Market volatility index
SHIBOR: Shanghai interbank offered rate
LIBOR: London interbank offered rate
FFR: U.S. federal fund benchmark rate
ORJ: Japanese benchmark rate
NLP: Natural language processing
RMSE: Root mean square error
MAE: Mean absolute error
MedAE: Median absolute error
GBDT: Gradient boosting decision tree
LightGBM: Light gradient boosting machine
MACD: Moving average convergence divergence
A/D: Accumulation/distribution
LWR: Larry Williams ratio
RSI: Relative strength index
SMA: Simple moving average
WMA: Weighted moving average
CCI: Commodity channel index

## References

[1] Abu N, Gamal AAM, Sakanko MA, Mateen A, Joseph D, Amaechi BOO (2021). How have COVID-19 confirmed cases and deaths affected stock markets? Evidence from Nigeria. Contemp. Econ..

[2] Alonso-Monsalve S, Suárez-Cetrulo AL, Cervantes A, Quintana D (2020). Convolution on neural networks for high-frequency trend prediction of cryptocurrency exchange rates using technical indicators. Expert Syst. Appl..

[3] Aloui D (2021). The COVID-19 pandemic haunting the transmission of the quantitative easing to the exchange rate. Finance Res. Lett..

[4] Amat C, Michalski T, Stoltz G (2018). Fundamentals and exchange rate forecastability with simple machine learning methods. J. Int. Money Financ..

[5] Bollerslev T (1986). Generalized autoregressive conditional heteroskedasticity. J. Econometric..

[6] Boubaker H, Zorgati MBS, Bannour N (2021). Interdependence between exchange rates: evidence from multivariate analysis since the financial crisis to the COVID-19 crisis. Econ. Anal. Policy.

[7] Bui LT, Truong Vu V, Huong Dinh TT (2018). A novel evolutionary multiobjective ensemble learning approach for forecasting currency exchange rates. Data Knowl. Eng..

[8] Cakici N, Zaremba A (2021). Who should be afraid of infections? Pandemic exposure and the cross-section of stock returns. J. Int. Finan. Markets. Inst. Money.

[9] Chandra R, Chand S (2016). Evaluation of co-evolutionary neural network architectures for time series prediction with mobile application in finance. Appl. Soft Comput..

[10] Chen, K., Zhou, Y., Dai, F.: A LSTM-based method for stock returns prediction: a case study of China stock market. In: 2015 IEEE international conference on big data (big data), pp. 2823–2824. IEEE (2015).

[11] Chong E, Han C, Park FC (2017). Deep learning networks for stock market analysis and prediction: methodology, data representations, and case studies. Expert Syst. Appl..

[12] Dash R (2017). An improved shuffled frog leaping algorithm based evolutionary framework for currency exchange rate prediction. Physica A Stat. Mech. Appl..

[13] Ding W, Levine R, Lin C, Xie W (2021). Corporate immunity to the COVID-19 pandemic. J. Financ. Econ..

[14] Evans C, Pappas K, Xhafa F (2013). Utilizing artificial neural networks and genetic algorithms to build an algo-trading model for intra-day foreign exchange speculation. Math. Comput. Model..

[15] Feng GF, Yang HC, Gong Q, Chang CP (2021). What is the exchange rate volatility response to COVID-19 and government interventions?. Econ. Anal. Policy.

[16] Fu S, Li Y, Sun S, Li H (2019). Evolutionary support vector machine for RMB exchange rate forecasting. Physica A Stat. Mech. Appl..

[17] Garg B, Prabheesh KP (2021). The nexus between the exchange rates and interest rates: evidence from BRIICS economies during the COVID-19 pandemic. Stud. Econ. Financ..

[18] Guo W, Chen Z, Šević A (2021). The political pressure from the US upon RMB exchange rate. J. Int. Finan. Markets. Inst. Money.

[19] Hiransha M, Gopalakrishnan EA, Menon VK, Soman KP (2018). NSE stock market prediction using deep-learning models. Procedia Comput. Sci..

[20] Hornik K, Stinchcombe M, White H (1989). Multilayer feedforward networks are universal approximators. Neural Netw..

[21] Hoshikawa T, Yoshimi T (2021). The effect of the COVID-19 pandemic on South Korea's stock market and exchange rate. Dev. Econ..

[22] Huang S-C, Chuang P-J, Wu C-F, Lai H-J (2010). Chaos-based support vector regressions for exchange rate forecasting. Expert Syst. Appl..

[23] Ince H, Trafalis TB (2006). A hybrid model for exchange rate prediction. Decis. Support Syst..

[24] Kara Y, AcarBoyacioglu M, Baykan OK (2011). Predicting direction of stock price index movement using artificial neural networks and support vector machines: the sample of the Istanbul stock exchange. Expert Syst. Appl..

[25] Ke, G., Meng, Q., Finley, T., Wang, T., Chen, W., Ma, W., Ye, Q., Liu, T.-Y.: LightGBM: a highly efficient gradient boosting decision tree. Adv. Neural Inf. Process. Syst. **30** (2017)

[26] Kiani KM, Kastens TL (2008). Testing forecast accuracy of foreign exchange rates: predictions from feed forward and various recurrent neural network architectures. Comput. Econ..

[27] Konstantakis KN, Melissaropoulos IG, Daglis T, Michaelides PG (2021). The euro to dollar exchange rate in the Covid-19 era: evidence from spectral causality and Markov-switching estimation. Int. J. Financ. Econ..

[28] Kristjanpoller W, Minutolo MC (2018). A hybrid volatility forecasting framework integrating GARCH, artificial neural network, technical analysis and principal components analysis. Expert Syst. Appl..

[29] Lahmiri S (2017). Modeling and predicting historical volatility in exchange rate markets. Physica A Stat. Mech. Appl..

[30] Liu TR, Gerlow ME, Irwin SH (1994). The performance of alternative VAR models in forecasting exchange rates. Int. J. Forecast..

[31] Mazur M, Dang M, Vega M (2021). COVID-19 and the March 2020 stock market crash Evidence from S&P1500. Financ. Res. Lett..

[32] Narayan PK (2021). Understanding exchange rate shocks during COVID-19. Financ. Res. Lett..

[33] Neely CJ (2009). Forecasting foreign exchange volatility: Why is implied volatility biased and inefficient? And does it matter?. J. Int. Finan. Markets. Inst. Money.

[34] Ni L, Li Y, Wang X, Zhang J, Yu J, Qi C (2019). Forecasting of forex time series data based on deep learning. Procedia Comput. Sci..

[35] NjindanIyke B (2020). The disease outbreak channel of exchange rate return predictability: Evidence from COVID-19. Emerg. Mark. Financ. Trade.

[36] NjindanIyke BN, Ho SY (2021). Exchange rate exposure in the South African stock market before and during the COVID-19 pandemic. Financ. Res. Lett..

[37] Pai P-F, Chen S-Y, Huang C-W, Chang Y-H (2010). Analyzing foreign exchange rates by rough set theory and directed acyclic graph support vector machines. Expert Syst. Appl..

[38] Panopoulou E, Souropanis I (2019). The role of technical indicators in exchange rate forecasting. J. Empir. Financ..

[39] Qureshi F (2021). COVID-19 pandemic, economic indicators and sectoral returns: evidence from US and China. Economic Research-Ekonomska Istraživanja.

[40] Rai K, Garg B (2021). Dynamic correlations and volatility spillovers between stock price and exchange rate in BRIICS economies: evidence from the COVID-19 outbreak period. Appl. Econ. Lett..

[41] Rehman MU, Al Rababa'a AR, El-Nader G, Alkhataybeh A, Vo XV (2022). Modelling the quantile cross-coherence between exchange rates: Does the COVID-19 pandemic change the interlinkage structure?. J. Int. Finan. Markets. Inst. Money.

[42] Ren Y, Liang X, Wang Q (2021). Short-term exchange rate forecasting: a panel combination approach. J. Int. Finan. Markets. Inst. Money.

[43] Selvin, S., Vinayakumar, R., Gopalakrishnan, E.A., Menon, V.K., Soman, K.P.: Stock price prediction using LSTM, RNN and CNN-sliding window model. In: 2017 international conference on advances in computing, communications and informatics (icacci), pp. 1643–1647. IEEE (2017)

[44] Shahzad SJH, Kyei CK, Gupta R, Olson E (2021). Investor sentiment and dollar-pound exchange rate returns: evidence from over a century of data using a crossquantilogram approach. Finance Res. Lett..

[45] Waheeb W, Ghazali R, Hussain AJ (2018). Dynamic ridge polynomial neural network with Lyapunov function for time series forecasting. Appl. Intell..

[46] Wang W, Su C, Duxbury D (2021). Investor sentiment and stock returns: global evidence. J. Empirical Finance.

[47] Wu Y, Gao J (2018). Adaboost-based long short-term memory ensemble learning approach for financial time series forecasting. Curr. Sci..

[48] Yu L, Lai KK, Wang S (2008). Multistage RBF neural network ensemble learning for exchange rates forecasting. Neurocomputing.

[49] Yu L, Wang S, Lai KK (2005). A novel nonlinear ensemble forecasting model incorporating GLAR and ANN for foreign exchange rates. Comput. Oper. Res..

[50] Zainuddin NH, Lola MS, Djauhari MA, Yusof F, Ramlee MNA, Deraman A, Abdullah MT (2019). Improvement of time forecasting models using a novel hybridization of bootstrap and double bootstrap artificial neural networks. Appl. Soft Comput. J..

[51] Zaremba A, Kizys R, Tzouvanas P, Aharon DY, Demir E (2021). The quest for multidimensional financial immunity to the COVID-19 pandemic: evidence from international stock markets. J. Int. Finan. Markets. Inst. Money.

[52] Zhang Y, Hamori S (2020). The predictability of the exchange rate when combining machine learning and fundamental models. J. Risk Financ. Manag..

[53] Zhang D, Hu M, Ji Q (2020). Financial markets under the global pandemic of COVID-19. Financ. Res. Lett..

[54] Zhang Y-Q, Wan X (2007). Statistical fuzzy interval neural networks for currency exchange rate time series prediction. Appl. Soft Comput. J..

